# The relationship between socioeconomic status/income and prevalence of diabetes and associated conditions: A cross-sectional population-based study in Saskatchewan, Canada

**DOI:** 10.1186/s12939-015-0237-0

**Published:** 2015-10-12

**Authors:** Yelena Bird, Mark Lemstra, Marla Rogers, John Moraros

**Affiliations:** School of Public Health, University of Saskatchewan, 104 Clinic Place, E-Wing Health Sciences, Room 3320, Saskatoon, SK S7N 5E5 Canada; College of Medicine, University of Saskatchewan, Saskatoon, Canada

**Keywords:** Diabetes, Associated conditions, Socioeconomic status, Income, Canada

## Abstract

**Introduction:**

The role that socioeconomic status/income play in accounting for the increased prevalence of type 2 diabetes has not been sufficiently studied in Canada. The primary purpose of the present study was to determine the unadjusted and adjusted effect of income on type 2 diabetes. The secondary purpose was to determine the adjusted effect of income on diabetes associated conditions such as high blood pressure and being overweight or obese, and its main behavioral factor of physical inactivity.

**Methods:**

This is a cross-sectional, population-based study. Data was analyzed from four cycles of the Canadian Community Health Survey (CCHS). It was conducted by Statistics Canada and covered the time period of 2000–2008 in the province of Saskatchewan, Canada. In this study, four separate and distinct multivariate models were built to determine the independent effect of income on type 2 diabetes and the associated conditions of high blood pressure, being overweight or obese, and physical inactivity.

**Results:**

The total sample size was comprised of 27,090 residents from Saskatchewan. After statistically controlling for age, only six covariates were independently associated with type 2 diabetes prevalence including: having high blood pressure (OR = 3.26), visible minority cultural status (OR = 2.17), being overweight or obese (OR = 1.97), being of male gender (OR = 1.76), having a household income of $29,999 per year (OR = 1.63) and being physically inactive (OR = 1.15).

**Conclusions:**

In this study, household income was strongly and independently associated with type 2 diabetes prevalence, its associated conditions of high blood pressure and being overweight or obese, and its main behavioral factor of physical inactivity. We suggest that income is an important but frequently overlooked factor for type 2 diabetes and worthy of further investigation, appropriate public debate and timely policy intervention.

## Introduction

Diabetes is considered to be the world’s fastest growing chronic disease. In 2013, there were 382 million people living with diabetes worldwide and the figure is expected to rise to 592 million by 2035. Globally, it is estimated that every six seconds, someone dies from diabetes related complications. In 2013, diabetes caused 5.1 million deaths and 548 billion dollars (USD) in healthcare expenditures alone [1].

Similar trends have been observed in Canada. In 2009, the Canadian Diabetes Association (CDA) estimated that the number of Canadians diagnosed diabetes will increase from 1.3 million in 2000 to 2.5 million in 2010 and 3.7 million in 2020. In their report, the CDA concluded that life expectancy can be reduced by up to 15 years for those individuals affected by type 1 diabetes and 5 to 10 years for those with type 2 diabetes, making diabetes the seventh leading cause of death in Canada. In terms of morbidity, it has been reported that 42 % of kidney dialysis patients have diabetes, 70 % of non-traumatic limb amputations are the result of complications from diabetes and the disease is considered to be the leading cause of blindness [2]. From an economic perspective, the CDA suggested that the corresponding burden of diabetes will increase from $6.3 billion in 2000 to $12.2 billion in 2010 and $16.9 billion in 2020 [2].

In recent years, several major health reports have been published in Canada that critically examine diabetes and its associated conditions. In 2009, the CDA concluded that the major risk factors responsible for the significant increase in the incidence of diabetes in Canada include rising obesity levels, increasing sedentary lifestyles and a growing percentage of the population being of Aboriginal descent [2]. In 2011, the Public Health Agency of Canada (PHAC) published a comprehensive report on diabetes and concluded the main modifiable risk factors to be overweight and obesity, physical inactivity, unhealthy eating, and smoking. Non-modifiable risk factors included ethnicity and recent immigration [3]. In 2013, Health Canada reported the modifiable risk factors for diabetes to be high body mass index, unhealthy eating, physical inactivity and inability to manage blood pressure, cholesterol and glucose levels [4]. However, all three reports failed to mention the possible significance of income as a factor for diabetes.

This is not entirely surprising. Sir Geoffrey Rose drew a distinction between the causes of individual cases and the causes of patterns of incidence in a population. Underlying determinants of health help decide which diseases are common in a population because they set the incidence rates. Therefore, while it may be easier for major reports and diabetes preventive strategies to focus on the main risk factors of the disease, this approach is limiting as it does not truly address the root causes of the problem. Rose concluded that to tackle this paradox will entail that we adopt a more comprehensive view of ill-health. It will also force us to acknowledge that the primary determinants of disease are mainly social and economic in nature. Thus and by necessity, it will require solutions that need to address socio-economic inequities [5].

Complicating matters even further is the fact that as Sir Michael Hurst, President of the International Diabetes Federation, pointed out there is a misconception held to date in many countries of the world including parts of Canada that diabetes is “a disease of the wealthy.” [6] However, a meta-analysis reviewing socioeconomic factors and diabetes found that lower income increased the risk of developing diabetes by 40 % even after statistically controlling for clinical factors and risk behaviors [7]. Similar findings have been reported in developing [8] and industrialized countries in the world [9,10] and Canada is no exception. Among 98,298 Canadians, the prevalence of type 2 diabetes was estimated to be 9.1 % among people with lower income but only 2.2 % among individuals with higher income. After statistically controlling for other factors, lower income males were 94 % more likely to have type 2 diabetes while lower income females were 175 % more likely to have type 2 diabetes [11]. Another review of 491,083 Canadians reported similar findings with regard to the prevalence of diabetes when comparing lower income (9.1 %) to higher income (3.2 %) Canadians respectively [12]. These findings are important and help highlight the disproportionate burden of type 2 diabetes among socio-economically disadvantaged individuals and communities.

The primary purpose of the present study was to determine the unadjusted and adjusted effect of income on type 2 diabetes. The secondary purpose was to determine the adjusted effect of income on diabetes associated conditions such as high blood pressure and being overweight or obese, and its main behavioral factor of physical inactivity.

## Methods

### Data source

Data was analyzed from the Canadian Community Health Survey (CCHS). The CCHS is a cross-sectional survey that collects self-reported information related to health status, health care utilization and health determinants for the Canadian population. The CCHS questionnaire is developed by specialists from the academic field, the federal government and Statistics Canada. It relies upon a large sample of respondents and is designed to provide representative and reliable estimates. The primary uses of CCHS data are for the purposes of health surveillance and population health research [13].

### Sampling frame

The sampling frame spanned over five cycles and covered the time period of 2000–2008 in Saskatchewan, Canada. Cycle 1 was collected from 2000–2001, Cycle 2 was collected in 2003, Cycle 3 was collected in 2005, and Cycle 4 and 5 was collected in 2007 and 2008, respectively.

All Cycles were based on random digit, dialing telephone survey samples with computer assisted interviewing. CCHS exclude Registered Indians living on reserves, those living in institutions (i.e., penitentiaries) and full-time members of the Canadian Armed Forces and Royal Canadian Mounted Police. The appropriateness of pooling CCHS data over cycles to increase precision of estimates of independent risk indicators has been well established previously [14].

### Ethics statement

The survey was approved and conducted by Statistics Canada. Responding to this survey was voluntary.

### Study area, period and population

The study population was drawn from the province of Saskatchewan, Canada. Saskatchewan is a prairie province that economically relies on its mines and agriculture industries. In 2013, Saskatchewan was estimated to have a population of a little more than 1 million people [15]. The total sample size for this study consisted of 27,090 residents. The study sample was comparable and representative of Saskatchewan, Canada [16]. Table 1 provides the demographic characteristics of the study sample in comparison to the Saskatchewan population. In brief, the average age of the study participants was 52.6 years old. By age group, a little less than half of the sample (46.7 %) was between the ages of 20–49 years old. Females represented 55.9 % of the sample size. Most respondents were married 52.3 %, followed by 23.4 % being widowed, divorced or separated. Over half of the respondents had some post-secondary education 52.5 %. The average personal income was $23,931 and the average household income was $37,533. The prevalence of diabetes in Saskatchewan during the study period increased steadily from 5.7 % in 2000–2001 to 6.7 % in 2003, 7.4 % in 2005, and 8.4 % in 2007–2008 [13].

**Table 1:** Demographics of the study sample in comparison to Saskatchewan population

Variables	Study sample (%)	Saskatchewan census (%)
Age		
20 to 29 years	4,019 (14.8)	125,490 (17.8)
30 to 39 years	4,253 (15.7)	111,490 (15.8)
40 to 49 years	4,396 (16.2)	147,105 (20.8)
50 to 59 years	4,572 (16.9)	128,460 (18.2)
60 to 69 years	3,708 (13.7)	80,820 (11.5)
70 to 79 years	3,569 (13.2)	64,285 (9.1)
80 years or older	2,573 (9.5)	47,920 (6.8)
Gender		
Male	11,951 (44.1)	475,240 (49.1)
Female	15,139 (55.9)	492,915 (50.9)
Marital status		
Married	14,177 (52.3)	396,500 (47.3)
Common-law	1,503 (5.5)	57,535 (6.9)
Widowed/separated/divorced	6,344 (23.4)	127,510 (15.2)
Single/Never married	5,066 (18.7)	256,450 (30.6)
Cultural status		
Caucasian	24,126 (89.1)	822,875 (85.0)
Minority	2,964 (10.9)	145,280 (15.0)
Household income		
$29,999 or less	12,056 (44.5)	not available
$30,000 - $79,999	10,024 (37.0)	not available
$80,000 or more	855 (3.2)	not available
Missing	4,155 (15.3)	
Education level		
Less than secondary	7,453 (27.5)	231,730 (30.2)
High school graduate	5,404 (20.0)	205,495 (26.8)
Post-secondary	14,233 (52.5)	319,015 (41.6)

### Variables

In total, 178 demographic (i.e., age, gender, marital status, cultural status), socio-economic (i.e., household income, education), behavioral (i.e., physical inactivity, smoking, alcohol usage, consumption of fruits and vegetables), disease (i.e., diabetes), associated conditions (i.e., being overweight or obese, high blood pressure), other diseases (i.e., heart disease, mental health), life stress and access to health care related variables were available for analysis.

#### Disease - Type 2 diabetes

It is worthy to note that CCHS Cycles 1–5, do not include a self- report of the participants’ type of diabetes (i.e. type 1, type 2 or gestational). However, the present study used a validated CCHS algorithm to help differentiate the type 2 diabetic respondents [17].

#### Associated Conditions – Hypertension & Body Mass Index (BMI)

Hypertension was self-reported. BMI was calculated from self-reported height and weight and measured using two variables: normal weight (24.9 ≤ BMI) and overweight/obese (BMI ≥ 25 kg/m2).

#### Behavioral Factor – Physical Inactivity

Physical activity was calculated using the frequency and duration of respondents’ reported leisure time activities in the previous 3 months and the metabolic energy demand of each activity, which yielded the energy expenditure (EE). In this study, three categories of physical activity were considered: inactive (EE < 1.5); moderate (1.5 ≤ E < 3) and active (EE ≥ 3).

#### Income

Three groups of approximately equal sample size (low income: $29,999 per year or less, middle income: $30,000 to $79,999 per year, and high income: $80,000 or more per year) were established. The cut-off points for the middle income group were defined based on the concept of “income adequacy”, which is derived by taking into account the total household income and the household’s size [18].

### Statistical analysis

In this study, the data were analyzed using the SPSS version for Windows 10 software package. Four separate and distinct multivariate models were built to determine the independent effect of income on diabetes, high blood pressure, being overweight or obese, and physical inactivity. A hierarchal well-formulated step-wise modeling approach was used instead of a computer-generated stepwise algorithm. The unadjusted effect of each covariate was determined and then entered one step at a time based on changes in the −2 log likelihood and the Wald test. Confounding was tested by comparing the estimated coefficient of the outcome variable from models containing and not containing the covariates. Interaction was tested with product terms. R^2^ was used to determine the proportion of variance in the outcome variables as expressed by the knowledge of the explanatory variables but not as a measure of the appropriateness of the final models. Goodness-of-fit of the final models was assessed with the Hosmer-Lemeshow statistical test. [19,20]

## Results

### Summary

The primary study analysis used cross tabulations among 178 variables. Prior to controlling for other factors, there were 19 variables that initially had an unadjusted yet statistically significant association with diabetes prevalence. For example, 9.0 % of those who had a household income of $29,999 per year or less had diabetes, while 4.3 % of those who made between $30,000 and $79,999 per year and only 2.7 % of those who made more than $80,000 per year had diabetes. Only variables with a statistically significant association are shown in Table 2.

**Table 2:** Statistically significant unadjusted associations with diabetes

Variables	Prevalence of diabetes (%)	P-value
Demographics		
Age		.000
20 to 29 years	1.0	
30 to 39 years	1.7	
40 to 49 years	3.9	
50 to 59 years	7.5	
60 to 69 years	12.5	
70 to 79 years	13.7	
80 years or older	12.8	
Gender		.000
Male	7.7	
Female	6.5	
Marital status		.000
Married	6.8	
Common-law	4.2	
Widowed/separated/divorced	10.4	
Single/never married	4.3	
Cultural status		.000
Caucasian	6.8	
Visible minority	9.2	
Socioeconomic		
Household income		.000
Low income: $29,999 or less	9.0	
Middle income: $30,000 - $79,999	4.3	
High income: $80,000 or more	2.7	
Education level		.000
Less than secondary	11.3	
Secondary graduate	5.1	
Post-secondary/graduate	5.3	
Employment status		.000
Unemployed	12.6	
Part-time	5.1	
Full-time	3.9	
Own your own home		.000
Yes	6.6	
No	8.3	
Behaviors		
Daily Smoker (age group)		.000
Less than 10 years	1.4	
11–20 years	1.9	
21–30 years	4.0	
31–40 years	6.0	
41–50 years	11.5	
51–60 years	12.8	
Physical activity level		.000
Inactive	8.2	
Moderate	5.8	
Active	4.6	
Daily fruit and vegetable consumption		.000
Less than 5	6.0	
5 or more	7.5	
Associated Conditions		
BMI		.000
Overweight/obese	8.7	
Normal weight	3.8	
High blood pressure		.000
Yes	17.1	
No	4.1	
Co-Morbidities		
Heart disease		.000
Yes	19.1	
No	6.0	
Suffers the effects of a stroke		.000
Yes	22.4	
No	6.7	
Cancer		.000
Yes	15.5	
No	6.8	
Arthritis/Rheumatism		.000
Yes	12.7	
No	5.0	
Consulted a health professional		.000
Yes	7.3	
No	2.3	
Life stress		.000
Quite a bit or extreme	6.6	
A bit	6.0	
Not at all or very	8.3	

After statistically controlling for age, only six covariates had an independent and adjusted association with diabetes prevalence including: having high blood pressure (OR = 3.26), visible minority cultural status (OR = 2.17), being overweight or obese (OR = 1.97), male gender (OR = 1.76), having a household income of $29,999 or less per year (OR = 1.63) and being physically inactive (OR = 1.15). The results are found in Table 3.

**Table 3:** Independent and adjusted risk indicators of diabetes after controlling for age

Independent variable	OR	95 % confidence interval	P-value
High blood pressure	3.26	2.87 – 3.70	.000
Cultural status: Visible minority	2.17	1.80 – 2.63	.000
Body Mass Index: Overweight/obese	1.97	1.71 – 2.27	.000
Gender: Male	1.76	1.67 – 1.86	.000
Household income: $29,999 or less	1.63	1.44 – 1.85	.000
Physically inactive	1.15	1.06 – 1.24	.001

Reference categories:

High blood pressure – no; Cultural status – Caucasian; BMI – Normal weight; Female gender; Household income - $80,000 or more; Physically active

### The association variable of high blood pressure

When cross tabulating the diabetes associated conditions of high blood pressure by household income, it was discovered that 27.6 % of those who made $29,999 per year or less had high blood pressure, when compared to 15.4 % of those who made between $30,000 and $79,999 per year, and 8.5 % of those who made more than $80,000 per year had high blood pressure. After statistically controlling for age, there were five covariates that had an independent and adjusted association with high blood pressure prevalence. These included being overweight or obese (OR = 2.14), being a daily smoker (OR = 1.84), having a household income below $30,000 per year (OR = 1.52), being of male gender (OR = 1.26) and being physically inactive (OR = 1.11). The results are found in Table 4.

**Table 4:** Independent and adjusted risk indicators of high blood pressure after controlling for age

Independent variable	OR	95 % confidence interval	P-value
Body Mass Index: Overweight/obese	2.14	1.97 – 2.33	.000
Daily smoker	1.84	1.80 – 1.88	.000
Household income: $29,999 or less	1.52	1.41 – 1.63	.000
Gender: Male	1.26	1.16 – 1.36	.000
Physically inactive	1.11	1.06 – 1.17	.000

Reference categories:

BMI – Normal weight; Non-smoker; Household income - $80,000 or more; Female gender; Physically active

### The association variable of BMI

When cross tabulating the diabetes associated conditions of being overweight or obese (BMI ≥ 25 kg/m2) by household income, 65.1 % of those who made $29,999 per year or less were overweight or obese. By comparison, 59.8 % of those who made between $30,000 and $79,999 per year and 51.2 % of those who made more than $80,000 per year were overweight or obese. After statistically controlling for age, there were only five covariates that had an independent and adjusted association with being overweight or obese. In order of importance, they were: having a household income below $30,000 per year (OR = 1.90), not being a daily smoker (OR = 1.82), being of male gender (OR = 1.51), visible minority cultural status (OR = 1.37) and being physically inactive (OR = 1.17). The results are found in Table 5.

**Table 5:** Independent and adjusted risk indicators of being overweight or obese after controlling for age

Independent variable	OR	95 % confidence interval	P-value
Household income: $29,999 or less	1.90	1.85 – 1.95	.000
Non-smoker	1.82	1.79 – 1.85	.000
Gender: Male	1.51	1.48 – 1.54	.000
Cultural status: Visible minority	1.37	1.23 – 1.51	.000
Physically inactive	1.17	1.13 – 1.21	.001

Reference categories:

Household income – $80,000 and over; Smoker; Female gender; Cultural status – Caucasian; Physically active

### The association variable of physical inactivity

Cross tabulating the main behavioral factor of physical inactivity by household income, 60 % of those who made $29,999 per year, 49.5 % of those who made between $30,000 and $79,999 per year, and 47.5 % of those who made more than $80,000 per year were daily physically inactive. After statistically controlling for age, there were six covariates that had an independent and adjusted association with physical inactivity. These included visible minority cultural status (OR = 1.83), being overweight/obese (OR = 1.32), having less than secondary education (OR = 1.25), being male (OR-1.17), having a household income below $30,000 per year (OR = 1.15) and being a daily smoker (OR = 1.12). The results are found in Table 6.

**Table 6:** Independent and adjusted risk indicators of physical inactivity after controlling for age

Independent variable	OR	95 % confidence interval	P-value
Cultural status: Visible minority	1.83	1.73 – 1.93	.001
Body Mass Index: Overweight/obese	1.32	1.23 – 1.41	.000
Educational level: Less than secondary	1.25	1.19 – 1.31	.000
Gender: Male	1.17	1.09 – 1.26	.000
Household income: $29,999 or less	1.15	1.08 – 1.23	.000
Daily smoker	1.12	1.08 – 1.17	.000

Reference categories:

Cultural status – Caucasian; BMI – normal weight; Education level – Post-sec/graduate; Female gender; Household income - $80,000 or more; Non-smoker

### Regression models

The R^2^ for the four regression models were 0.212, 0.198, 0.191 and 0.141 respectively, suggesting reasonable explanation of the proportion of variance observed in this study. Similarly, the goodness-of-fit test results (p = 0.811, 0.871, 0.831, 0.772) suggest that the four models are appropriate and that the predicted values are accurate representations of the observed values in an absolute sense. Given the fact that the estimated slope coefficients and standard errors are small, co-linearity is not suspected.

## Discussion

The results of the present study show the prevalence of diabetes to be inversely and strongly related to household income. It was found that 9.0 % of those who had a household income of $29,999 per year or less had diabetes, when compared to 4.3 % of those who made between $30,000 and $79,999, and only 2.7 % of those who made more than $80,000. This is an unadjusted ratio of 3.33. However, after statistically adjusting for age and five other covariates, the adjusted odds ratio becomes 1.63. As such, part of the unadjusted association between income and diabetes can be attributed to the other covariates in the final regression model. Nonetheless, this still suggests that many cases of diabetes among low and middle-income residents in Saskatchewan, Canada may be preventable if equitable measures were taken to reduce their financial gap from higher income households. Income was also strongly and independently associated with diabetes associated conditions, namely high blood pressure and being overweight or obese, and its main behavioral factor, physical inactivity.

Our results are consistent with the ones reported in other studies. As mentioned previously, a large Canadian study found the prevalence of diabetes to be 9.1 % among lower income Canadians and 3.2 % among higher income Canadians [12]. The same study also reviewed high blood pressure by income quartile. From lowest to highest income group, the prevalence of high blood pressure was 15.4 %, 13.8 %, 9.8 % and 7.3 % respectively [12]. Similarly, high blood pressure was strongly associated with both lower income and higher prevalence of diabetes in our study. It is conceivable that the challenging living conditions experienced by those residing in poor neighborhoods makes it difficult for them to adhere to their high blood pressure treatment and access the healthcare resources required to bring their condition under control.

Obesity is also known to be a potent risk factor for the development of diabetes. In the majority of cities in Canada, obesity is more prevalent in the most socio-economically deprived neighborhoods. For example, in Halifax, 25.5 % of people in the low income areas were obese compared to 11.2 % of people residing in the high income areas [21]. The findings of our study provide further evidence in support of this link. However, lower income could also be the result of diabetes since its chronic nature and severe complications may limit employment and educational opportunities for those affected.

Statistics Canada reports physical inactivity levels to vary considerably between low income (58 %) and high income Canadians (36.5 %) [3]. This finding may very well be a function of the lack of infrastructure in poorer neighborhoods, which is known to be a barrier to physical activity. Reportedly, low income neighborhoods have fewer and less safe playgrounds and green spaces as well as general lack of accessibility to physical activity equipment, facilities and programs. [22] The lack of opportunities for physical activity in poor neighborhoods not only impacts obesity rates but as our study results show, may also help explain their association with diabetes.

The discussion of income as a key factor to develop diabetes is an important one because it is evident that the prevalence of the disease is rising disproportionately by level of income. In a national Canadian study over an eleven year period, the prevalence of diabetes increased by 56 % in the lowest income group, 93 % in the lower middle income group, 59 % in the upper middle income group and 0 % in the highest income group [12]. This finding becomes more meaningful when one considers that Canada’s population is not only aging [4] but the financial gap between its high income earners and the rest of the population is rapidly widening [23]. These developments have major implications on the management (i.e. health care utilization) and impact (i.e. morbidity and mortality rates) of chronic diseases such as diabetes.

Income is also known to affect health care utilization for diabetic patients. In a report from Saskatchewan, Canada, those who lived in low-income neighborhoods had age-standardized hospitalization rates for diabetes of 212 per 100,000 population in comparison to 16 per 100,000 for those in high income neighborhoods. Residents living in low-income neighborhoods had higher rates of overall physician visits (15,804 per 100,000 population) for diabetes in comparison to those living in the high income neighborhoods (7,456 per 100,000 population). Similarly, residents living in low-income neighborhoods also had higher rates of diabetes medication fills (42,903 per 100,000 population) in comparison to those living in high income neighborhoods (16,491 per 100,000 population) [24]. Income even impacts mortality rates among those with diabetes. A study conducted in Ontario reviewed all deaths from 1994 to 2005 and concluded that the age and sex adjusted mortality rate of diabetics between the highest and lowest income groups had widen by more than 40 % [25].

It is perhaps ironic that people in poor neighborhoods with the lowest levels of security in income are also most likely to develop diabetes, and once they do, they lack access to important resources to help them properly manage their disease. This mismatch between stress, and reduced capacity to deal effectively with distress, may help explain the higher rates of chronic disease in general and diabetes specifically observed among poor and vulnerable populations [26].

### Limitations and Strengths

The present study has a few limitations. Its design is cross-sectional in nature and it can only imply association but not causation. As such, the study design does not explain specifically how income impacts the prevalence of diabetes. The data are self-reported and therefore, many of the variables considered in this study may be under-reported. Additionally, the study did not use stratified analyses to help account for the different data collection periods. Finally, the findings of our study while applicable to the province of Saskatchewan may not be generalizable and therefore, one should be cautious about drawing conclusions at national or international levels.

In spite of these limitations, the present study has a number of significant strengths. It provides a sound analysis of the association between income and the prevalence of diabetes. More importantly, it helps elucidate the impact that income has on diabetes associated conditions, namely high blood pressure and being overweight or obese, and its main behavioral factor of physical inactivity. These findings provide much needed evidence and help explain the potential chain of events and adverse effects that low income may have on diabetes, its associated conditions and behavioral factors. The present study also helps highlight the fact that individuals with lower income not only suffer disproportionately from diabetes but may also be ill-equipped to adequately manage their disease.

Finally, while the study was limited to Saskatchewan, it is worthwhile noting that Saskatchewan is one of the Canadian provinces with the worst health outcomes on a number of diseases including diabetes. As such, this study and its findings provide concrete evidence and help fuel the ongoing public debate within the province of Saskatchewan, Canada, and internationally about the role income and by extension socio-economic status may play in causing higher morbidity and mortality rates due to diabetes and its associated conditions.

## Conclusions

To the authors’ knowledge, this is the first population-based study of its kind in Saskatchewan, Canada. Our study adds value to the growing international body of knowledge that inexorably links lower household income to higher diabetes rates. In summary, it was found that household income was strongly and independently associated with diabetes prevalence, its associated conditions of high blood pressure and being overweight or obese, and its main behavioral factor of physical inactivity. We suggest that income is an important but frequently overlooked factor for diabetes and worthy of further investigation, appropriate public debate and timely policy intervention.

## Abbreviations

CCHS: Canadian Community Health Survey
CDA: Canadian Diabetes Association
PHAC: Public Health Agency of Canada
RCMP: Canadian Armed Forces and Royal Canadian Mounted Police
USD: United States Dollar
